# Antioxidation and Cytoprotection of Acteoside and Its Derivatives: Comparison and Mechanistic Chemistry

**DOI:** 10.3390/molecules23020498

**Published:** 2018-02-23

**Authors:** Xican Li, Yulu Xie, Ke Li, Aizhi Wu, Hong Xie, Qian Guo, Penghui Xue, Yerkingul Maleshibek, Wei Zhao, Jiasong Guo, Dongfeng Chen

**Affiliations:** 1 School of Chinese Herbal Medicine, Guangzhou University of Chinese Medicine, Guangzhou 510006, China; xieyulu1900@163.com (Y.X.); xiehongxh1@163.com (H.X.); 15622178307@163.com (Q.G.); 15228738137@163.com (P.X.); pandiphd@163.com (Y.M.); 2 Innovative Research & Development Laboratory of TCM, Guangzhou University of Chinese Medicine, Guangzhou 510006, China; 3 School of Basic Medical Science, Guangzhou University of Chinese Medicine, Guangzhou 510006, China; ys1090992678@163.com (K.L.); chen888@gzucm.edu.cn (D.C.); 4 The Research Center of Basic Integrative Medicine, Guangzhou University of Chinese Medicine, Guangzhou 510006, China; 5 School of Basic Medical Science, Guangdong Pharmaceutical University, Guangzhou 510007, China; 6 Zhongshan School of Medicine, Sun Yat-sen University, No. 74 Zhongshan Road. 2, Guangzhou 510080, China; zhaowei23@mail.sysu.edu.cn; 7 Department of Histology and Embryology, Southern Medical University, Guangzhou 510515, China; jiasongguo@aliyun.com

**Keywords:** acteoside, apiosyl, forsythoside B, phenylpropanoid glycosides, poliumoside, rhamnosyl

## Abstract

The study tried to explore the role of sugar-residues and mechanisms of phenolic phenylpropanoid antioxidants. Acteoside, along with its apioside forsythoside B and rhamnoside poliumoside, were comparatively investigated using various antioxidant assays. In three electron-transfer (ET)-based assays (FRAP, CUPRAC, PTIO•-scavenging at pH 4.5), the relative antioxidant levels roughly ruled as: acteoside >forsythoside B > poliumoside. Such order was also observed in H^+^-transfer-involved PTIO•-scavenging assay at pH 7.4, and in three multiple-pathway-involved radical-scavenging assays, i.e., ABTS^+^•-scavenging, DPPH•-scavenging, and •O_2_^−^-scavenging. In UV-vis spectra, each of them displayed a red-shift at 335→364 nm and two weak peaks (480 and 719 nm), when mixed with Fe^2+^; however, acteoside gave the weakest absorption. In Ultra-performance liquid chromatography coupled with electrospray ionization quadrupole time-of-flight tandem mass spectrometry (UPLC−ESI−Q−TOF−MS/MS) analysis, no radical-adduct-formation (RAF) peak was found. MTT assay revealed that poliumoside exhibited the highest viability of oxidative-stressed bone marrow-derived mesenchymal stem cells. In conclusion, acteoside, forsythoside B, and poliumoside may be involved in multiple-pathways to exert the antioxidant action, including ET, H^+^-transfer, or Fe^2+^-chelating, but not RAF. The ET and H^+^-transfer may be hindered by rhamnosyl and apiosyl moieties; however, the Fe^2+^-chelating potential can be enhanced by two sugar-residues (especially rhamnosyl moiety). The general effect of rhamnosyl and apiosyl moieties is to improve the antioxidant or cytoprotective effects.

## 1. Introduction

**Figure 1:**
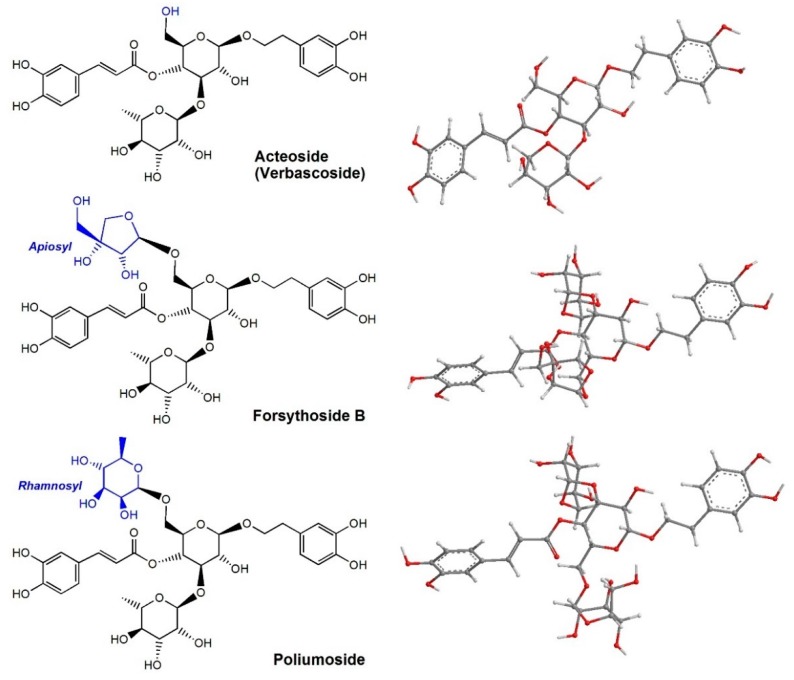
The structures (**left**) and preferential-conformation-based ball-and-stick models (**right**) of three natural phenylpropanoid glycosides (acteoside, forsythoside B, and poliumoside).

Recently, acteoside (verbascoside, Figure 1), a phenolic phenylpropanoid glycoside occurring in vervain and other plants [1], was found to improve the efficiency of canine somatic cell nuclear transfer (SCNT) during the dog-cloning process [2]. In addition, acteoside could protect human neuroblastoma SH-SY5Y cells against β-amyloid-induced damage [3] and human skin fibroblasts against X-ray-induced damage [4]. Its apioside forsythoside B (Figure 1) is also distributed in plants [5], and has been observed to exert potent neuroprotective effects with a favorable therapeutic time-window [6]. These beneficial effects on cells and tissues are thought to be associated with the protection of some biomolecules, such as lipids and DNA. In fact, acteoside and its analog *cis*-tanoside F have been demonstrated to inhibit mitochondrial lipid peroxidation of rats [7]. Lipid peroxidation is known to be a result of cellular oxidative stress and ultimately from reactive oxygen species (ROS).

From a DNA protection aspect, acteoside and its derivatives can quickly repair DNA radicals, such as 2’-deoxyadenosine-5’-monophosphate (dAMP) and 2’-deoxyguanosine-5’-monophosphate (dGMP) [8]. These secondary deoxynucleotide radicals can further oxidatively damage cells leading to mutagenesis and carcinogenesis [9]. Biophysical study indicated that acteoside and its derivatives could dock into DNA minor grooves and quickly repair such oxidative DNA damage [10]. The preferential-conformation-based ball-stick model proposes (Figure 1, right) that these molecules are prolate and can easily reach the loop of DNA, and thus, they are able to quickly repair the damage of DNA radicals [8]. 

However, from the viewpoint of biochemistry, the repair is essentially fulfilled via an ROS-scavenging (i.e., antioxidant) pathway. As previously mentioned, the generation of deoxynucleotide radical cations are ultimately from the attack of ROS, including free radicals (e.g., •OH radical [11]) and oxidant molecules (e.g., H_2_O_2_ [12]). ROS-scavenging is thought to efficiently lower cellular oxidative status [13,14] and improve the quality of stem cells [12,15]. Therefore, it is necessary to study their antioxidant ability and further discuss possible mechanisms.

In this study, three phenolic phenylpropanoid glycosides (acteoside, forsythoside B, and poliumoside) were comparatively investigated using various antioxidant assays, including the 2-phenyl-4,4,5,5-tetramethylimidazoline-1-oxyl-3-oxide radical (PTIO•) scavenging assay, ferric ion reducing antioxidant power (FRAP) assay, cupric reducing antioxidant capacity (CUPRAC) assay, 2,2′-azino-bis(3-ethylbenzo-thiazoline-6-sulfonic acid radical (ABTS^+^•) scavenging assay, 1,1-diphenyl-2-picryl-hydrazl (DPPH•) assay, and •O_2_^−^-scavenging assay as well as Fe^2+^-chelating UV-spectra analysis. Subsequently, their reaction products with DPPH• were determined using ultra-performance liquid chromatography coupled with electrospray ionization quadrupole time-of-flight tandem mass spectrometry (UPLC−ESI−Q−TOF−MS/MS) analysis. Finally, their cytoprotective effect towards bone marrow-derived mesenchymal stem cells (bmMSCs) was estimated using the 3-(4,5-dimethylthiazol-2-yl)-2,5-diphenyl (MTT) assay. As seen in Figure 1, the difference among the three phenolic phenylpropanoid glycosides (i.e., acteoside, forsythoside B, and poliumoside) is the glycoside type: forsythoside B is an apioside of acteoside, while poliumoside is a rhamnoside of acteoside. Thus, this comparative study also helps in understanding the roles of apiosyl and rhamnosyl in antioxidant effects.

## 2. Results

### 2.1. Metal-Reducing Assays (FRAP & CUPRAC)

In the study, we carried out two metal-reducing assays, i.e., FRAP assay and CUPRAC assay. As illustrated in Suppl. 1, acteoside and its derivatives increased the relative FRAP (or Cu^2+^-reducing power) percentages at 0–10 μg/L in a dose-dependent manner. Their order of relative metal-reducing levels roughly decreased in the order of acteoside > forsythoside B > poliumoside (Table 1).

### 2.2. PTIO•-Scavenging Assay

The PTIO•-scavenging assay was recently developed by our laboratory [16]. It can be used to evaluate the antioxidant levels and to explore the antioxidant pathways. As shown in Suppl. 1, acteoside and its derivatives could successfully scavenge PTIO• at pH 4.5 and pH 7.4. However, according to the IC_50_ values in Table 1, their relative PTIO•-scavenging levels at the same pH value were different from each other. Also, each of acteoside and its derivatives exhibited different PTIO•-scavenging levels between pH 4.5 and pH 7.4. In general, the PTIO•-scavenging levels at 7.4 were higher than those at pH 4.5.

### 2.3. ABTS^+^•-Scavenging and DPPH•-Scavenging Assays

The ABTS^+^•-scavenging and DPPH•-scavenging are now widely used for the antioxidant studies of pure compounds or extracts. As seen in Suppl. 1, acteoside and its derivatives concentration-dependently increased the ABTS^+^•-scavenging or DPPH•-scavenging percentages. However, in terms of the IC_50_ values in Table 1, their relative ABTS^+^•-scavenging levels were found in the order of: acteoside > forsythoside B > poliumoside > Trolox. A similar order could also be observed in the DPPH•-scavenging assay.

### 2.4. UPLC−ESI−Q−TOF−MS/MS Analysis of DPPH• Reaction Products

The reaction product of DPPH• with each of three phenylpropanoid glycosides was explored using UPLC−ESI−Q−TOF−MS/MS analysis. In the analysis (Suppl. 2), none of the three phenylpropanoid glycosides produced RAF product peak. By comparison, caffeic acid was found to yield a dimer product (*m*/*z* 359–360, Suppl. 2).

### 2.5. UV-Vis-Spectra Analysis of Fe^2+^-Chelating Products

**Figure 2:**
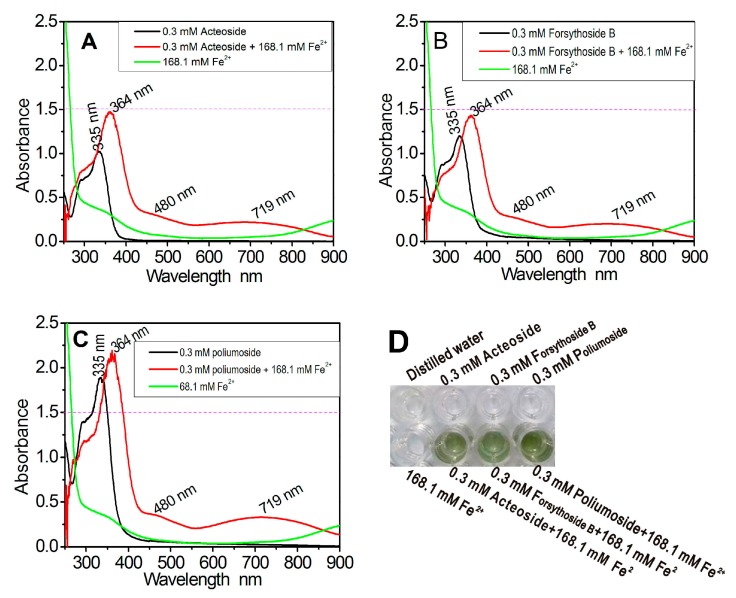
The results of Fe^2+^-chelating reaction of acteoside and its derivatives. (**A**) UV-vis spectra of the reaction mixture of Fe^2+^ with acteoside; (**B**) UV-vis spectra of the reaction mixture of Fe^2+^ with forsythoside B; (**C**) UV-vis spectra of the reaction mixture of Fe^2+^ with poliumoside; (**D**) The solution appearances.

Since UV-vis-spectra can characterize metal complexes, the study thus used UV-vis-spectra to analyze the possible Fe^2+^-chelating reaction with three phenylpropanoid glycosides. As shown in Figure 2, each of them could produce a bathochromic shift (335 nm→364 nm) in the UV spectra. Meanwhile, each of them could provide two weak vis-spectra peaks in the visible spectra (480 nm and 719 nm) and leading to a light green appearance. However, the peak intensity was in a descending order of poliumoside > forsythoside B > acteoside.

### 2.6. Pyrogallol Autooxidation Assay for Superoxide Anion (•O_2_^−^) Scavenging 

The pyrogallol autooxidation assay was improved by our laboratory in 2012 [17]. It was used to estimate the •O_2_^−^-scavenging potential in the present study. As seen in Suppl. 1, all of acteoside and its derivatives could dose-dependently increase the •O_2_^−^-scavenging percentages. However, the relative bioactivity decreased in the order of poliumoside > forsythoside B > acteoside, according to the IC_50_ values in Table 1.

### 2.7. Cytoprotective Effect towards Oxidatively Stressed bmMSCs (MTT Assay)

To assess the cytoprotective effects of acteoside and its derivatives, we performed a MTT assay. In the assay, bmMSCs were damaged by oxidative reagent H_2_O_2_, the damaged bmMSCs were then treated by acteoside and its derivatives. The A_490nm_ values was used to assess the relative cytoprotective effects. As seen in Table 2, each of acteoside and its derivatives dose-dependently increased the A_490nm_ values. However, at the same concentration, poliumoside gave the highest A_490nm_ values.

## 3. Discussion

The antioxidant action of natural phenolic compounds is known to be involved in electron-transfer (ET) [18,19]. Thus, some ET-based metal-reducing assays have been widely used to assess antioxidant levels of phenolics, such as the FRAP and CUPRAC assays. The FRAP assay guidelines are to be fulfilled with a pH less than 3.6. Such an acidic environment has successfully suppressed H^+^ ionization from phenolics; thus, the FRAP assay is considered as a mere ET process [20,21]. The effectiveness of acteoside and its derivatives in the FRAP assay implies that, when acteoside and its derivatives act as antioxidants, they may use the ET pathway to exert their antioxidant action.

Besides, we also performed a CUPRAC assay in a pH 7.4 buffer. As seen in Suppl. 1, acteoside and its derivatives dose-dependently increased their Cu^2+^-reducing power percentages, indicating that they could remain ET potential at physiological pH. However, their ET potentials decreased in the following order: acteoside > forsythoside B > poliumoside (Table 1). This dynamic clearly suggests that apiosyl moiety in forsythoside B and rhamnosyl moiety in poliumoside lowered the ET potential.

To test the possibility that ET occurs during their radical-scavenging processes, an oxygen-centered free radical PTIO• was introduced in the study. Cyclic voltammetry evidence revealed that PTIO•-scavenging below pH 5.0 is a single electron-redox reaction [22]. The observation that acteoside and its derivatives could efficiently scavenge the PTIO• radical at pH 4.5, suggests the possibility of ET during their radical-scavenging processes. Obviously, this finding further supports the aforementioned results from FRAP and CUPRAC assays, and previous results that a donating electron (e) is a feature of phenolic antioxidants [23].

At physiological pH 7.4, however, the PTIO•-scavenging assay is not merely an ET pathway but also includes a proton- (H^+^) transfer pathway. During the process, PTIO• has been suggested to accept H^+^ from phenolics to produce the product peak ([PTIO-H]^+^) [16]. Because H^+^-transfer is always accompanied by ET in stepwise or synchronous mechanisms [24], the realistic (or final) product is a [PTIO-H] molecule [22]. The PTIO•-scavenging at pH 7.4 (Suppl. 1) implies that acteoside and its derivatives possess an H^+^-transfer potential as well. The IC_50_ values (Table 1) indicated that the relative H^+^-transfer potentials were in a descending order of acteoside > forsythoside B > poliumoside. Clearly, the apiosyl and rhamnosyl moieties also weakened the H^+^-transfer potential during the antioxidant process. 

As previously discussed, during the antioxidant process of phenolics, ET is usually accompanied by proton (H^+^) transfer to form several antioxidant mechanisms [24], such as hydrogen-atom transfer (HAT) [23,25,26,27], sequential electron-proton transfer (SEPT) [26,27], sequential proton loss single-electron transfer (SPLET) [26], and proton-coupled electron transfer (PCET) [24,25,26,28]. For example, ABTS^+^•-scavenging, a reaction dominated by single-electron transfer (SET) [29], has also been proven to be affected by H^+^ levels recently [30]. ABTS^+^•-scavenging is therefore a multi-pathway-based antioxidant assay [21,31]. The fact that acteoside and its derivatives could scavenge ABTS^+^• radicals indicates that their antioxidant action may also be mediated via multi-pathways. This hypothesis is further confirmed by the evidence from the DPPH•-scavenging assay, a reaction comprising HAT, ET, SEPT, and PCET multiple pathways [26,32]. However, the quantitative analysis based IC_50_ values (Table 1) revealed that, in multi-pathway-based ABTS^+^•-scavenging and DPPH•-scavenging aspects, acteoside was superior to its apioside forsythoside B and rhamnoside poliumoside. Thus, it can be deduced that apiosyl and rhamnosyl moieties eventually hinder multi-pathway potentials (especially ET and H^+^-transfer) during the free-radical-scavenging process.

As noted by the authors and others [14,26], during the antioxidant process, an RAF reaction may also occur. To verify the RAF possibility, however, three phenylpropanoid glycosides along with caffeic acid were studied using UPLC−ESI−Q−TOF−MS/MS analysis. Caffeic acid was found to yield a dimer product, while three phenylpropanoid glycosides produced no peak of RAF product. This finding clearly suggests that three phenylpropanoid glycosides cannot undergo RAF pathway to exert their antioxidant action. Since three phenylpropanoid glycosides can be regarded as the esters of caffeic acid (Figure 1), such a difference between caffeic acid and caffeic acid esters also indicates that huge moiety may hinder the generation of RAF.

Taken together, from a free-radical-scavenging aspect, acteoside and its derivatives may undergo multiple pathways to exert their antioxidant action. These antioxidant pathways at least are involved in ET and H^+^-transfer (but not RAF). Our findings are partly supported by the theoretical study that acteoside could exert the antioxidant action via SPLET pathway. In the process, acteoside might firstly deprotonate (H^+^-transfer) to yield anion. The deprotonation is thought to occur in the catechol moieties with weak acidity. Subsequently, the anion donated electron to give rise to phenoxy radical form [33]. Phenoxy radical with p-π conjugation however is stable to some extent. Of course, in this respect, further experimental work is needed in the future.

It is worth mentioning that cellular oxidative stress can also originate from transition metals (especially Fe^2+^). The Fe^2+^ ion, however, can transform the H_2_O_2_ molecule into a most harmful •OH radical via the Fenton reaction (Fe^2+^ + H_2_O_2_ → Fe^3+^ + •OH + OH^−^). Therefore, attenuation of Fe^2+^ levels can effectively inhibit •OH radicals to release cellular oxidative stress. In fact, iron-chelating by natural phenolic antioxidants now has been developed into an effective therapy for some oxidative-stress diseases [34,35].

In the present study, acteoside and its derivatives were suggested as effective Fe^2+^-chelators by the changes in spectroscopy and solution colors (Figure 2). Nevertheless, acteoside is inferior to the two glucosides in chelating Fe^2+^ and that forsythoside B with apiosyl moiety is inferior to poliumoside with rhamnosyl moiety. Based on the comparison of their preferential conformations (Figure 1, right), it is proposed that apiosyl (or rhamnosyl) moiety can aid the main ligand (phenylpropanoic group) in chelating Fe^2+^. Such a synergistic effect undoubtedly strengthens the Fe^2+^-chelating ability and enlarges the UV-vis peaks. However, rhamnosyl is more effective than apiosyl in its Fe^2+^-chelating ability. The difference can be attributed to the fact that rhamnosyl occurs in a hexocyclic form (i.e., α-l-rhamnopyranosyl), while apiosyl is in a pentacyclic form (i.e., β-d-apiofuransyl). A hexocyclic form is known to be larger and more stable. Hence, hexocyclic rhamnosyl is more effective as compared to pentacyclic apiosyl in its Fe^2+^-chelating ability.

To test whether acteoside and its derivatives can scavenge ROS, we conducted a pyrogallol autoxidation assay. As seen in Suppl. 1, all could efficiently scavenge the •O_2_^−^ radical, a typical ROS occurring in cells. However, the relative bioactivity decreased in the order poliumoside > forsythoside B > acteoside. This order is also parallel to that of the cytoprotective effects (Table 2). This finding indicates that the general effect of rhamnosyl moiety or apiosyl moiety is to enhance ROS-scavenging or cytoprotective effects.

## 4. Materials and Methods

### 4.1. Chemicals and Animals

Acteoside (CAS number: 61276-17-3, 97%), forsythoside B (CAS number: 81525-13-5, 97%) were obtained from BioBioPha (Kunming, China, Suppl. 3). Poliumoside (CAS number: 94079-81-9, 97%) was isolated by our team from the traditional Chinese herb *Callicarpa peii* H.T. Chang (Suppl. 3). The DPPH•, (±)-6-hydroxyl-2,5,7,8-tetramethlychromane-2-carboxylic acid (Trolox), 2,9-dimethyl-1,10-phenanthroline (neocuproine), 2,4,6-tripyridyltriazine (TPTZ), and pyrogallol were purchased from Sigma-Aldrich Shanghai Trading Co. (Shanghai, China). (NH_4_)_2_ABTS [2,2′-azino-bis (3-ethylbenzo-thiazoline-6-sulfonic acid diammonium salt)] was obtained from Amresco Chemical Co. (Solon, OH, USA). PTIO• radical was purchased from TCI Development Co., Ltd. (Shanghai, China). Caffeic acid was purchased from National Institute for the Control of Pharmaceutical and Biological Products (Beijing, China). Dulbecco’s modified Eagle’s medium (DMEM), fetal bovine serum (FBS), and trypsin were purchased from Gibco (Grand Island, NY, USA). Annexin V/propidium iodide (PI) assay kit was purchased from Invitrogen (Carlsbad, CA, USA). All other reagents were of analytical grade.

Sprague-Dawley (SD) rats of 4 weeks of age were obtained from the Animal Center of Guangzhou University of Chinese Medicine. The protocol of this experiment was performed under the supervision of the Institutional Animal Ethics Committee in Guangzhou University of Chinese (Approval number 20170306A).

### 4.2. Metal-Reducing Assays (FRAP & CUPRAC)

Metal-reducing assays include the Fe^3+^-reducing power assay and Cu^2+^-reducing power assay. The Fe^3+^-reducing assay was established by Benzie and Strain and is formally named as FRAP [20]. The experimental protocol of this assay was described in a previous report [9]. Briefly, the FRAP reagent was prepared freshly by mixing 10 mM TPTZ, 20 mM FeCl_3_ and 0.25 M acetate buffer at a ratio of 1:1:10 at pH 3.6. The test sample (x = 4–20 μL, 0.05 mg/mL) was added to (20 − x) μL of 95% ethanol followed by 80 μL of FRAP reagent. After a 30-min incubation at ambient temperature, the absorbance was measured at 595 nm using a microplate reader (Multiskan FC, Thermo Scientific, Shanghai, China). The relative reducing power of the sample was calculated using the following formula:

(1) $$Relative\;reducing\;effect\;\%=\frac{A-A_{\min}}{A_{\max}-A_{\min}}\times 100\%$$

where A_max_ was the maximum absorbance of the reaction mixture with sample, and A_min_ is the minimum absorbance in the test. A is the absorbance of the sample.

Cu^2+^-reducing power can also characterize antioxidant level and thus is termed CUPRAC. This assay was carried out according to a previously published method [36]. Briefly, 12 μL of CuSO_4_ aqueous solution (10 mmol/L), 12 μL of neocuproine ethanolic solution (7.5 mmol/L) and (75 − x) μL of CH_3_COONH_4_ buffer solution (0.1 mol/L, pH 7.5) were added to wells with different volumes of sample (0.05 mg/mL, 4–20 μL). The absorbance at 450 nm after 30 min was measured using the aforementioned microplate reader. The relative CUPRAC power was calculated using the formula for FRAP. A_max_ was the maximum absorbance of the reaction mixture with sample; and A_min_ is the minimum absorbance in the test. A is the absorbance of the sample. 

### 4.3. PTIO•-Scavenging Assay

The PTIO•-scavenging assays (at pH 4.5 or pH 7.4) were conducted based on our method [16]. In brief, the test sample solution (x = 0–20 μL, 1 mg/mL for pH 4.5 and 0.5 mg/mL for pH 7.4) was added to (20 − x) μL of 95% ethanol, followed by 80 μL of an aqueous PTIO• solution. The aqueous PTIO• solution was prepared using a phosphate-butter solution (0.1 mM, pH 4.5 or pH 7.4). The mixture was maintained at 37 °C for 2 h, and the absorbance was then measured at 560 nm using the aforementioned microplate reader. The PTIO• inhibition percentage was calculated as follows:

(2) $$\mathrm{Inhibition}\%=\frac{A_{0}-A}{A_{0}}\times 100\%$$

where A_0_ is the absorbance of the control without the sample, and A is the absorbance of the reaction mixture with the sample.

### 4.4. ABTS^+^•-Scavenging and DPPH•-Scavenging Assays

The ABTS•^+^-scavenging activity was evaluated according to the method [37]. The ABTS^+^• was produced by mixing 0.2 mL of ABTS diammonium salt (7.4 mmol/L) with 0.2 mL of potassium persulfate (2.6 mmol/L). The mixture was kept in the dark at room temperature for 12 h to allow completion of radical generation before being diluted with distilled water (at a ratio of approximately 1:20) so that its absorbance at 734 nm was 0.35 ± 0.01 using the aforementioned microplate reader. To determine the scavenging activity, the test sample (x = 4–20 μL, 0.05 mg/mL) was added to (20 − x) μL of distilled water followed by 80 μL of ABTS^+^• reagent, and the absorbance at 734 nm was measured 3 min after initial mixing, using distilled water as the blank.

DPPH• radical-scavenging activity was determined as previously described [18]. Briefly, 75 μL of DPPH• solution (0.1μM) was mixed with the indicated concentrations of the sample (0.025 mg/mL, 5–25 μL) dissolved in methanol. The mixture was maintained at room temperature for 30 min, and the absorbance was measured at 519 nm using the aforementioned microplate reader. 

The percentages of ABTS^+^•-scavenging activity and DPPH•-scavenging activity were calculated based on the formula presented in Section 4.3.

### 4.5. UPLC−ESI−Q−TOF−MS/MS Analysis of DPPH• Reaction Products 

This method was based on our previous study [25]. The methanol solution of acteoside was mixed with a solution of DPPH• radicals in methanol at a molar ratio of 1:2, and the resulting mixture was incubated for 24 h at room temperature. The product mixture was then filtered through a 0.22-μm filter and analyzed using a UPLC system equipped with a C_18_ column (2.0 mm i.d. × 100 mm, 1.6 µm, Phenomenex, Torrance, CA, USA). The mobile phase was used for the elution of the system and consisted of a mixture of methanol (phase A) and water (phase B). The column was eluted at a flow rate of 0.3 mL/min with the following gradient elution program: 0–10 min, 60–100% A; 10–15 min, 100%A. The sample injection volume was set at 1 μL for the separation of the different components. ESI-Q-TOF-MS/MS analysis was performed using a Triple TOF 5600 *plus* Mass spectrometer (AB SCIEX, Framingham, MA, USA) equipped with an ESI source, which was run in the negative ionization mode. The scan range was set at 100–2000 Da. The system was run with the following parameters: ion spray voltage, −4500 V; ion source heater, 550 °C; curtain gas (CUR, N2), 30 psi; nebulizing gas (GS1, Air), 50 psi; Tis gas (GS2, Air), 50 psi. The declustering potential (DP) was set at −100 V, whereas the collision energy (CE) was set at −40 V with a collision energy spread (CES) of 20 V. The RAF products were quantified by extracting the corresponding molecular formula from the total ion chromatogram (Suppl. 2).

The aforementioned experiment was repeated using forsythoside B, poliumoside, and caffeic acid. The corresponding *m*/*z* peaks were extracted from the corresponding molecular formula from the total ion chromatogram (Suppl. 2). 

### 4.6. UV-Vis-Spectra Analysis of Fe^2+^-Chelating Products

The Fe^2+^-chelating reaction products of acteoside-Fe^2+^ were evaluated using UV-Vis-spectroscopy [13]. For the experiment, 300 μL of a methanolic solution of acteoside (0.24 mM) was added to 700 μL of an aqueous solution of FeCl_2_·4H_2_O (168 mM). The solution was then mixed vigorously. Subsequently, the resulting mixture was scanned using a UV-Vis spectrophotometer after an hour (Unico 2600A, Shanghai, China) from 200–850 nm. 

The aforementioned experiment was repeated using forsythoside B, or poliumoside, instead of acteoside.

### 4.7. Pyrogallol Autooxidation Assay for Superoxide Anion (•O_2_^−^) Scavenging

Measurement of superoxide anion (•O_2_^−^) scavenging activity was based on our method [17]. Briefly, the sample was dissolved in ethanol at 1 mg/mL. The sample solution (x μL) was mixed with Tris-HCl buffer (980 − x μL, 0.05 M, pH 7.4) containing EDTA (1 mM). When 20 μL pyrogallol (60 mM in 1 mM HCl) was added, the mixture was shaken at room temperature immediately. The absorbance at 325 nm of the mixture was measured (Unico 2100, Shanghai, China) against the Tris-HCl buffer as a blank every 30 s for 5 min. The •O_2_^−^ scavenging ability was calculated as follows:

(3) $$\mathrm{Inhibition}\%=\frac{\left(\frac{\Delta A_{325\mathrm{nm},\mathrm{control}}}{T}\right)-\left(\frac{\Delta A_{325\mathrm{nm},\mathrm{sample}}}{T}\right)}{\left(\frac{\Delta A_{325\mathrm{nm},\mathrm{control}}}{T}\right)}\times 100\%$$

Here, ΔA_325nm_, control is the increment in A_325nm_ of the mixture without the sample, and ΔA_325nm_, sample is the increment in A_325nm_ of the mixture with the sample; T = 5 min. The experimental temperature was 37 °C.

### 4.8. Cytoprotective Effect Towards Oxidatively Stressed bmMSCs (MTT Assay) 

The bmMSCs were cultured according to our previous reports [38] with slight modifications. In brief, bone marrow was obtained from the femur and tibia of a rat. The marrow samples were diluted with DMEM (low glucose) containing 10% FBS. The bmMSCs were prepared by gradient centrifugation at 900 g for 30 min at 1.073 g/mL Percoll. The prepared cells were detached by treatment with 0.25% trypsin and passed into cultural flasks at 1 × 104/cm^2^. The bmMSCs at passage 3 were evaluated for cultured cell homogeneity using detection of CD44 using MTT assay [39].

**Figure 3:**
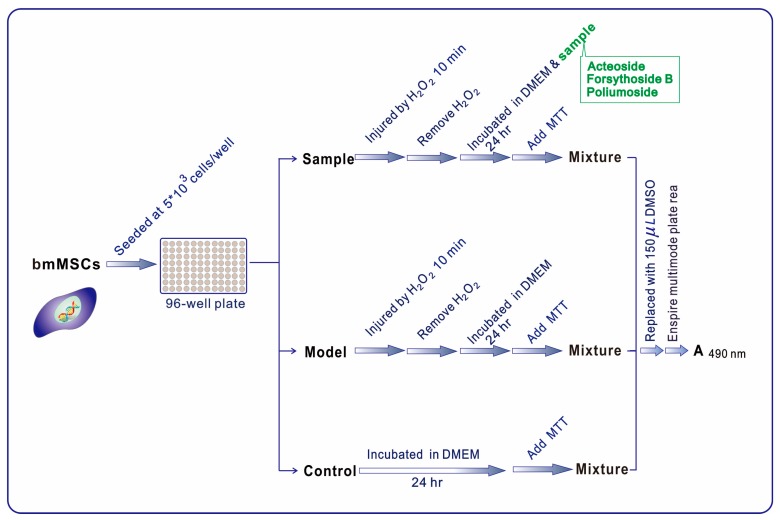
Flow chart of MTT assay experiment (Bio-Kinetics reader was the product of PE-1420; Bio-Kinetics Corporation, Sioux Center, IA, USA). MTT was at 5 mg/mL (in PBS), and the addition volume was 20 µL. Addition of oxidative reagent was conducted by injection of H_2_O_2_ (50 μM).

The MTT assay was used to evaluate the cytoprotective effect of acteoside and its derivatives towards bmMSCs [40]. The injury model was established based on the previous study [41]. The experimental protocol is briefly illustrated in Figure 3.

### 4.9. Statistical Analysis

Each experiment in Section 4.2, Section 4.3, Section 4.4 and Section 4.7 was performed in triplicate, and the MTT assay experiment was performed in pentaplicate. Data were recorded as the mean ± SD (standard deviation). The dose response curves were plotted using Origin 6.0 professional software (OriginLab, Northampton, MA, USA). The IC_50_ value was defined as the final concentration of 50% radical inhibition (relative reducing power) [42]. Statistical comparisons were made by one-way ANOVA to detect significant differences using SPSS 13.0 (SPSS Inc., Chicago, IL, USA) for Windows. *p* < 0.05 was considered to be statistically significant.

## 5. Conclusions

Three natural phenylpropanoid glycosides, namely, acteoside, forsythoside B, and poliumoside, can be involved in multiple pathways to exert antioxidant action, including ET, H^+^-transfer, and Fe^2+^-chelating, but not RAF. The ET and H^+^-transfer pathways may be hindered by rhamnosyl moiety or apiosyl moiety; however, the Fe^2+^-chelating pathway can be enhanced by sugar residues (especially rhamnosyl moiety). The general effect of rhamnosyl moiety or apiosyl moiety is to enhance multiple-pathway-involved ROS-scavenging ability. Thus, forsythoside B and poliumoside are superior to acteoside in cytoprotective effects. 

## Figures and Tables

**Table 1:** The IC_50_ values of acteoside, forsythoside B and poliumoside in various antioxidants assays.

Assays	Acteoside μM	Forsythoside B μM	Poliumoside μM	Trolox μM
FRAP	5.4 ± 0.6 ^a^	7.7 ± 0.2 ^c^	8.1 ± 0.2 ^d^	6.8 ± 0.4 ^b^
CUPRAC	4.8 ± 0.4 ^a^	6.1 ± 0.5 ^b^	6.5 ± 0.3 ^c^	7.8 ± 0.2 ^d^
PTIO•-scavenging (pH 4.5)	247.3 ± 21.2 ^b,B^	352.2 ± 20.8 ^c,B^	219.5 ± 10.4 ^b,B^	164.0 ± 7.5 ^a^
PTIO•-scavenging (pH 7.4)	120.8 ± 2.5 ^a,A^	125.5 ± 6.5 ^b,A^	127.2 ± 2.1 ^c,A^	223.7 ± 6.5 ^d^
ABTS^+^•-scavenging	12.5 ± 1.9 ^a^	14.0 ± 2.6 ^b^	19.9 ± 1.7 ^c^	25.8 ± 4.8 ^d^
DPPH•-scavenging	7.6 ± 0.1 ^a^	8.7 ± 0.1 ^b^	10.9 ± 0.6 ^c^	24.2 ± 0.3 ^d^
•O_2_^−^-scavenging	731.0 ± 1.7 ^b^	262.6 ± 3.3 ^a^	266.3 ± 4.6 ^a^	1205.2 ± 19.8 ^c^

The IC_50_ value (in μg/mL unit) was defined as the final concentration of 50% radical inhibition or relative reducing power and was calculated by linear regression analysis and expressed as the mean ± SD (n = 3). The linear regression was analyzed using Origin 6.0 professional software. The IC_50_ value (in μg/mL unit) was conversed into that of an μM unit and collected in brackets. The IC_50_ value in the μM unit with different superscripts (a, b, c, or d) in the same row are significantly different (*p <* 0.05); The IC_50_ value in the μM unit in PTIO assay with different superscripts (A or B) are significantly different (*p* < 0.05) between at pH 4.5 and pH 7.4. Trolox is the positive control. The dose-response curves are listed in Suppl. 1.

**Table 2:** The A_490nm_ of acteoside and its derivatives towards H_2_O_2_-damaged MSCs in MTT assay.

Groups	Acteoside	Forsythoside B	Poliumoside
Control	0.64 ± 0.05	0.64 ± 0.05	0.64 ± 0.05
Model	0.07 ± 0.01	0.07 ± 0.01	0.07 ± 0.01
10 μg/mL	0.07 ± 0.01	0.08 ± 0.01	0.10 ± 0.01
30 μg/mL	0.09 ± 0.01 ^a^	0.09 ± 0.01 ^a^	0.13 ± 0.01 ^b,^*
50 μg/mL	0.09 ± 0.01 ^a^	0.11 ± 0.01 ^b,^*	0.14 ± 0.01 ^c,^*
100 μg/mL	0.13 ± 0.01 ^a,^*	0.18 ± 0.01 ^b,^*	0.24 ± 0.01^c,^*

Experiments were performed with three different batches of cells and each batch was tested in triplicate. bmMSCs, bone marrow-derived mesenchymal stem cells; Each value is expressed as the mean ± SD, n = 3; * *p* < 0.05 vs. model. The values different superscripts (a, b, or c) in the same row are significantly different (*p* < 0.05).

## Supplementary Materials

Supplementary materials are available online. 1. Dose response curves; 2. HPLC-MS spectra; 3. Analysis certificates of acteoside, forsythoside B and poliumoside.

## Abbreviations

ABTS^+^•	2,2′-azino-bis (3-ethylbenzo-thiazoline-6-sulfonic acid) radical
bmMSCs	bone marrow-derived mesenchymal stem cells
CUPRAC	cupric reducing antioxidant capacity
dAMP	2’-deoxyadenosine-5’-monophosphate radical
DMEM	Dulbecco’s modified Eagle’s medium
dGMP	2’-deoxyguanosine-5’-monophosphate radical
DPPH•	1,1-diphenyl-2-picryl-hydrazl radical
ET	electron transfer; FBS: Fetal bovine serum
FRAP	ferric ion reducing antioxidant power;
HAT	hydrogen atom transfer
MTT	3-(4,5-dimethylthiazol-2-yl)-2,5-diphenyl
PCET	proton-coupled electron transfer
PTIO•	2-phenyl-4,4,5,5-tetramethylimidazoline-1-oxyl 3-oxide radical
RAF	radical adduct formation
ROS	reactive oxygen species
SCNT	somatic cell nuclear transfer
SEPT	sequential electron proton transfer
SPLET	sequential proton loss single electron transfer
TPTZ	2,4,6-tripyridyl triazine
Trolox	(±)-6-hydroxyl-2,5,7,8-tetramethlychromane-2-carboxylic acid
