# Supplementary material for: Antioxidation and Cytoprotection of Acteoside and Its Derivatives: Comparison and Mechanistic Chemistry
Source: Molecules. 2018 Feb 23;23(2):498. doi: 10.3390/molecules23020498 (PMC6017589; doi:10.3390/molecules23020498)
Supplement: Supplementary file 1 [file molecules-23-00498-s001.zip › Suppls/Suppl. 1 Dose response curves.docx]

**Suppl. 1-** **Dose response curves**

**Antioxidation and Cytoprtection of Acteoside and its Derivatives: Comparison and Mechanistic Chemistry**

Xican Li ^1,2,^ *^,†^, Yulu Xie ^1,2,†^, Ke Li ^3,4^, Aizhi Wu ^1,2,^ *, Hong Xie ^1,2^, Qian Guo ^1,5^, Penghui Xue ^1^, Yerkingul Maleshibek ^1^, Wei Zhao ^6^, Jiasong Guo ^7^, and Dongfeng Chen ^3,4^

^1^ School of Chinese Herbal Medicine; Guangzhou University of Chinese Medicine, Guangzhou 510006, China. E-mails: xieyulu1900@163.com (Y.X.); xiehongxh1@163.com (H.X.); 15622178307@163.com (Q.G.); 15228738137@163.com (P.X.); pandiphd@163.com (Y.M.);

^2^ Innovative Research & Development Laboratory of TCM; Guangzhou University of Chinese Medicine, Guangzhou 510006, China.

^3^ School of Basic Medical Science, Guangzhou University of Chinese Medicine, Guangzhou, China, 510006; E-mails: [ys1090992678@163.com](mailto:ys1090992678@163.com) (K.L.)

^4^ The Research Center of Basic Integrative Medicine, Guangzhou University of Chinese Medicine, Guangzhou, China, 510006. E-mail: chen888@gzucm.edu.cn (D.C.)

^5^ School of Basic Medical Science; Guangdong Pharmaceutical University, Guangzhou, China, 510007.

^6^ Zhongshan School of Medicine; Sun Yat-sen University, No.74 Zhongshan Road. 2, Guangzhou, 510080, China.

^7^ Department of Histology and Embryology, Southern Medical University, Guangzhou, 510515, China.

^*^Correspondence author:

E-mail：[lixican@126.com](mailto:lixican@126.com) [wuaizhi@gzucm.edu.cn](mailto:wuaizhi@gzucm.edu.cn)

Homepage:[www.researchgate.net/profile/Xican_Li](http://www.researchgate.net/profile/Xican_Li)

**



**

**Figure S1.** Relative metal-reducing power of acteoside and its derivatives: A, FRAP assay; B, CUPRAC assay. Each value is expressed as the mean±SD (n=3)

**
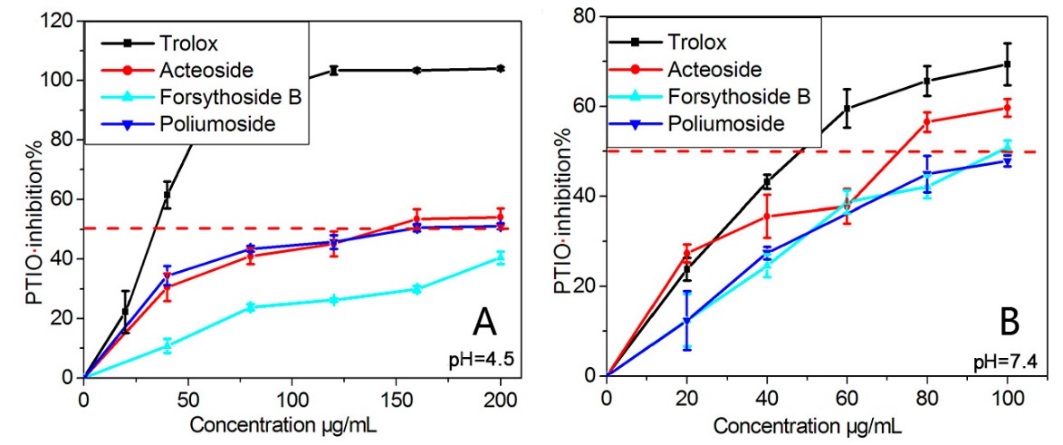
**

**Figure S2.** PTIO•-scavenging abilities of acteoside and its derivatives: A, pH 4.5; B, pH 7.4 Each value is expressed as the mean±SD (n=3)

**
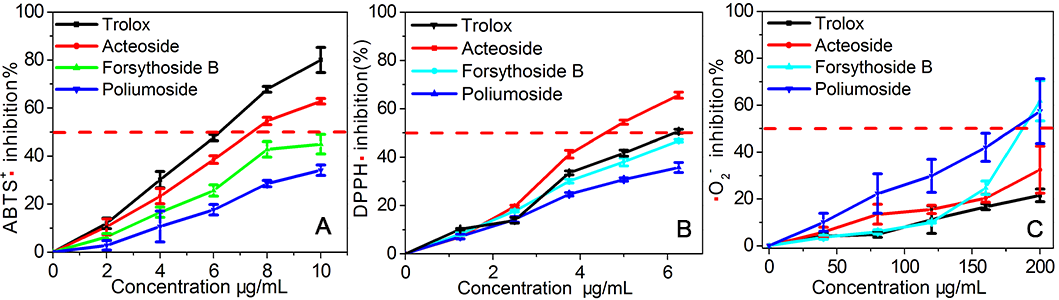
**

**Figure S3.** The effects of acteoside and its derivative scavenge in ABTS•^+^-scavenging (A), DPPH•^+^-scavenging (B), and (C) •O_2_^-^-scavenging. Each value is expressed as the mean±SD (n=3)
