# Supplementary material for: Antioxidation and Cytoprotection of Acteoside and Its Derivatives: Comparison and Mechanistic Chemistry
Source: Molecules. 2018 Feb 23;23(2):498. doi: 10.3390/molecules23020498 (PMC6017589; doi:10.3390/molecules23020498)
Supplement: Supplementary file 1 [file molecules-23-00498-s001.zip › Suppls/Suppl. 2 Original spectra of HPLC-MS spectra.docx]

**Suppl. 2.** **Original spectra of HPLC-MS spectra**

**Antioxidation and Cytoprotection of Acteoside and its Derivatives: Comparison and Mechanistic Chemistry**

Xican Li ^1,2,^ *^,†^, Yulu Xie ^1,2,†^, Ke Li ^3,4^, Aizhi Wu ^1,2,^ *, Hong Xie ^1,2^, Qian Guo ^1,5^, Penghui Xue ^1^, Yerkingul Maleshibek ^1^, Wei Zhao ^6^, Jiasong Guo ^7^, and Dongfeng Chen ^3,4^

^1^ School of Chinese Herbal Medicine; Guangzhou University of Chinese Medicine, Guangzhou 510006, China. E-mails: xieyulu1900@163.com (Y.X.); xiehongxh1@163.com (H.X.); 15622178307@163.com (Q.G.); 15228738137@163.com (P.X.); pandiphd@163.com (Y.M.);

^2^ Innovative Research & Development Laboratory of TCM; Guangzhou University of Chinese Medicine, Guangzhou 510006, China.

^3^ School of Basic Medical Science, Guangzhou University of Chinese Medicine, Guangzhou, China, 510006; E-mails: [ys1090992678@163.com](mailto:ys1090992678@163.com) (K.L.)

^4^ The Research Center of Basic Integrative Medicine, Guangzhou University of Chinese Medicine, Guangzhou, China, 510006. E-mail: chen888@gzucm.edu.cn (D.C.)

^5^ School of Basic Medical Science; Guangdong Pharmaceutical University, Guangzhou, China, 510007.

^6^ Zhongshan School of Medicine; Sun Yat-sen University, No.74 Zhongshan Road. 2, Guangzhou, 510080, China.

^7^ Department of Histology and Embryology, Southern Medical University, Guangzhou, 510515, China.

^*^Correspondence author:

E-mail：[lixican@126.com](mailto:lixican@126.com) [wuaizhi@gzucm.edu.cn](mailto:wuaizhi@gzucm.edu.cn)

Homepage:[www.researchgate.net/profile/Xican_Li](http://www.researchgate.net/profile/Xican_Li)

Note:

The supplemental materials are involved in four compounds, i.e. acteoside, forsythoside B, poliumoside, and caffeic acid. These materials tried to demonstrate that, each of acteoside, forsythoside B, and poliumoside have no RAF reaction with DPPH•, and no dimerization reaction; However, caffeic acid happen a dimerization reaction.

The determining conditions are detailed in Section 4.5 of main text.

Fig. S2.1 [Total ion chromatogram](http://www.baidu.com/link?url=CF2kwqGsxR0oJevz0eMOkaKlSp5m3CP5azTuAPtPYQywgNd79FAccvr91aMdqMonPznhus0HQnaImc9imgKjcZGfDHgKz2a0BLH0fQFGHU8hiKdjXP7HKk5Oo13Brtbt) of **acteoside**

Fig. S2.2 [Total ion chromatogram](http://www.baidu.com/link?url=CF2kwqGsxR0oJevz0eMOkaKlSp5m3CP5azTuAPtPYQywgNd79FAccvr91aMdqMonPznhus0HQnaImc9imgKjcZGfDHgKz2a0BLH0fQFGHU8hiKdjXP7HKk5Oo13Brtbt) of DPPH•

Fig. S2.3 [Ion chromatogram of](http://www.baidu.com/link?url=CF2kwqGsxR0oJevz0eMOkaKlSp5m3CP5azTuAPtPYQywgNd79FAccvr91aMdqMonPznhus0HQnaImc9imgKjcZGfDHgKz2a0BLH0fQFGHU8hiKdjXP7HKk5Oo13Brtbt)  reaction product of **acteoside** with DPPH• extracted by C_47_H_46_N_5_O_21_

The reaction was conducted by mixing acteoside and DPPH• (1:2, molar ratio) . The reaction mixture was incubated for 24h. The product mixture was analyzed using UPLC−ESI−Q−TOF−MS/MS technology. For the convenience of determination, the corresponding chemical formula of acteoside-DPPH• adduct was extracted by the software. The corresponding chemical formula was C_47_H_46_N_5_O_21_ (acteoside is C_29_H_36_O_15_; DPPH• is C_18_H_12_N_5_O_6._ Thus, [covalent](http://www.baidu.com/link?url=_NXpqH1Jy8-wYHBhYKz4OjCAE3JoN555TWPHLpcBZLLZRIaurfOQAmGBjgiSpGmKGQT_S1R2aAwmy80CmAIxxeLdfEfJ5extKEgaPUc1uAm) adduct should be C_47_H_46_N_5_O_21_). However, the spectra give no corresponding peak (The intensity in [Y-axis](http://www.baidu.com/link?url=Z6HvnDoXbWTkMqe9qMuC1GgntWV4Hi8GWrpEWlWrktwYOlPO_3DubKxLBZb7zWwWqIqkHfSl0rRVa4LyjMMtjvX_Qg7AKe1m3InrGmMl2Au) is negligible)

Fig. S2.4 [Ion chromatogram of](http://www.baidu.com/link?url=CF2kwqGsxR0oJevz0eMOkaKlSp5m3CP5azTuAPtPYQywgNd79FAccvr91aMdqMonPznhus0HQnaImc9imgKjcZGfDHgKz2a0BLH0fQFGHU8hiKdjXP7HKk5Oo13Brtbt)  reaction product of **acteoside** dimer extracted by C_58_H_70_O_30_

The reaction and determination conditions were described in the footnote of Fig. S2.3. For the convenience of determination, the corresponding chemical formula of acteoside-acteoside dimer was extracted by the software. The corresponding chemical formula was C_58_H_70_O_30_ (acteoside is C_29_H_36_O_15._ Thus, dimeric acteoside [covalent](http://www.baidu.com/link?url=_NXpqH1Jy8-wYHBhYKz4OjCAE3JoN555TWPHLpcBZLLZRIaurfOQAmGBjgiSpGmKGQT_S1R2aAwmy80CmAIxxeLdfEfJ5extKEgaPUc1uAm) adduct should be C_58_H_70_O_30_). However, the spectra give no corresponding peak. (The intensity in [Y-axis](http://www.baidu.com/link?url=Z6HvnDoXbWTkMqe9qMuC1GgntWV4Hi8GWrpEWlWrktwYOlPO_3DubKxLBZb7zWwWqIqkHfSl0rRVa4LyjMMtjvX_Qg7AKe1m3InrGmMl2Au) is negligible)

Fig. S2.5 [Total ion chromatogram](http://www.baidu.com/link?url=CF2kwqGsxR0oJevz0eMOkaKlSp5m3CP5azTuAPtPYQywgNd79FAccvr91aMdqMonPznhus0HQnaImc9imgKjcZGfDHgKz2a0BLH0fQFGHU8hiKdjXP7HKk5Oo13Brtbt) of **forsythoside B**

Fig. S2.6 [Total ion chromatogram](http://www.baidu.com/link?url=CF2kwqGsxR0oJevz0eMOkaKlSp5m3CP5azTuAPtPYQywgNd79FAccvr91aMdqMonPznhus0HQnaImc9imgKjcZGfDHgKz2a0BLH0fQFGHU8hiKdjXP7HKk5Oo13Brtbt) of DPPH•

Fig. S2.7 [Ion chromatogram of](http://www.baidu.com/link?url=CF2kwqGsxR0oJevz0eMOkaKlSp5m3CP5azTuAPtPYQywgNd79FAccvr91aMdqMonPznhus0HQnaImc9imgKjcZGfDHgKz2a0BLH0fQFGHU8hiKdjXP7HKk5Oo13Brtbt)  reaction product of **forsythoside B** with DPPH• extracted by C_52_H_54_N_5_O_25._ The reaction was conducted by mixing acteoside and DPPH• (1:2, molar ratio) . The reaction mixture was incubated for 24h. The product mixture was analyzed using UPLC−ESI−Q−TOF−MS/MS technology. For the convenience of determination, the corresponding chemical formula of forsythoside B-DPPH• adduct was extracted by the software. The corresponding chemical formula was C_52_H_54_N_5_O_25_ (forsythoside B is C_34_H_44_O_19_; DPPH• is C_18_H_12_N_5_O_6._ Thus, [covalent](http://www.baidu.com/link?url=_NXpqH1Jy8-wYHBhYKz4OjCAE3JoN555TWPHLpcBZLLZRIaurfOQAmGBjgiSpGmKGQT_S1R2aAwmy80CmAIxxeLdfEfJ5extKEgaPUc1uAm) adduct should be C_52_H_54_N_5_O_25_). However, the spectra give no corresponding peak. (The intensity in [Y-axis](http://www.baidu.com/link?url=Z6HvnDoXbWTkMqe9qMuC1GgntWV4Hi8GWrpEWlWrktwYOlPO_3DubKxLBZb7zWwWqIqkHfSl0rRVa4LyjMMtjvX_Qg7AKe1m3InrGmMl2Au) is negligible)

Fig. S2.8 [Ion chromatogram of](http://www.baidu.com/link?url=CF2kwqGsxR0oJevz0eMOkaKlSp5m3CP5azTuAPtPYQywgNd79FAccvr91aMdqMonPznhus0HQnaImc9imgKjcZGfDHgKz2a0BLH0fQFGHU8hiKdjXP7HKk5Oo13Brtbt)  reaction product of **forsythoside B** dimer extracted by C_68_H_86_O_38_.

The reaction and determination conditions were described in the footnote of Fig. S2.7. However, the chemical formula C_68_H_86_O_38_ was extracted for detection of forsythoside B-forsythoside B dimer (forsythoside B is C_34_H_44_O_19._ Thus, the dimer should be C_68_H_86_O_38_). However, the spectra give no corresponding peak. (The intensity in [Y-axis](http://www.baidu.com/link?url=Z6HvnDoXbWTkMqe9qMuC1GgntWV4Hi8GWrpEWlWrktwYOlPO_3DubKxLBZb7zWwWqIqkHfSl0rRVa4LyjMMtjvX_Qg7AKe1m3InrGmMl2Au) is negligible)

Fig. S2.9 [Total ion chromatogram](http://www.baidu.com/link?url=CF2kwqGsxR0oJevz0eMOkaKlSp5m3CP5azTuAPtPYQywgNd79FAccvr91aMdqMonPznhus0HQnaImc9imgKjcZGfDHgKz2a0BLH0fQFGHU8hiKdjXP7HKk5Oo13Brtbt) of **poliumoside**

Fig. S2.10 Total ion chromatogram of **DPPH•**

Fig. S2.11 [Ion chromatogram of](http://www.baidu.com/link?url=CF2kwqGsxR0oJevz0eMOkaKlSp5m3CP5azTuAPtPYQywgNd79FAccvr91aMdqMonPznhus0HQnaImc9imgKjcZGfDHgKz2a0BLH0fQFGHU8hiKdjXP7HKk5Oo13Brtbt)  reaction product of **poliumoside** with DPPH• extracted by C_52_H_54_N_5_O_25._ The reaction was conducted by mixing poliumoside and DPPH• (1:2, molar ratio) . The reaction mixture was incubated for 24h. The product mixture was analyzed using UPLC−ESI−Q−TOF−MS/MS technology. For the convenience of determination, the corresponding chemical formula of poliumoside-DPPH• adduct was extracted by the software. The corresponding chemical formula was C_53_H_56_N_5_O_25_ (poliumoside is C_35_H_46_O_19_; DPPH• is C_18_H_12_N_5_O_6._ Thus, [covalent](http://www.baidu.com/link?url=_NXpqH1Jy8-wYHBhYKz4OjCAE3JoN555TWPHLpcBZLLZRIaurfOQAmGBjgiSpGmKGQT_S1R2aAwmy80CmAIxxeLdfEfJ5extKEgaPUc1uAm) adduct should be C_53_H_56_N_5_O_25_). However, the spectra give no corresponding peak. (The intensity in [Y-axis](http://www.baidu.com/link?url=Z6HvnDoXbWTkMqe9qMuC1GgntWV4Hi8GWrpEWlWrktwYOlPO_3DubKxLBZb7zWwWqIqkHfSl0rRVa4LyjMMtjvX_Qg7AKe1m3InrGmMl2Au) is negligible)

Fig. S2.12 [Ion chromatogram of](http://www.baidu.com/link?url=CF2kwqGsxR0oJevz0eMOkaKlSp5m3CP5azTuAPtPYQywgNd79FAccvr91aMdqMonPznhus0HQnaImc9imgKjcZGfDHgKz2a0BLH0fQFGHU8hiKdjXP7HKk5Oo13Brtbt)  reaction product of **poliumoside** dimer extracted by C_70_H_90_O_38_.

The reaction and determination conditions were described in the footnote of Fig. S2.1. However, the chemical formula C_70_H_90_O_38_ was extracted for detection of poliumoside-poliumoside dimer (poliumoside is C_35_H_46_O_19._ Thus, the dimer should be C_70_H_90_O_38_). However, the spectra give no corresponding peak. (The intensity in [Y-axis](http://www.baidu.com/link?url=Z6HvnDoXbWTkMqe9qMuC1GgntWV4Hi8GWrpEWlWrktwYOlPO_3DubKxLBZb7zWwWqIqkHfSl0rRVa4LyjMMtjvX_Qg7AKe1m3InrGmMl2Au) is negligible)

Fig. S2.13 [Total ion chromatogram](http://www.baidu.com/link?url=CF2kwqGsxR0oJevz0eMOkaKlSp5m3CP5azTuAPtPYQywgNd79FAccvr91aMdqMonPznhus0HQnaImc9imgKjcZGfDHgKz2a0BLH0fQFGHU8hiKdjXP7HKk5Oo13Brtbt) of **caffeic acid**

Fig. S2.14 Total ion chromatogram of **DPPH•**


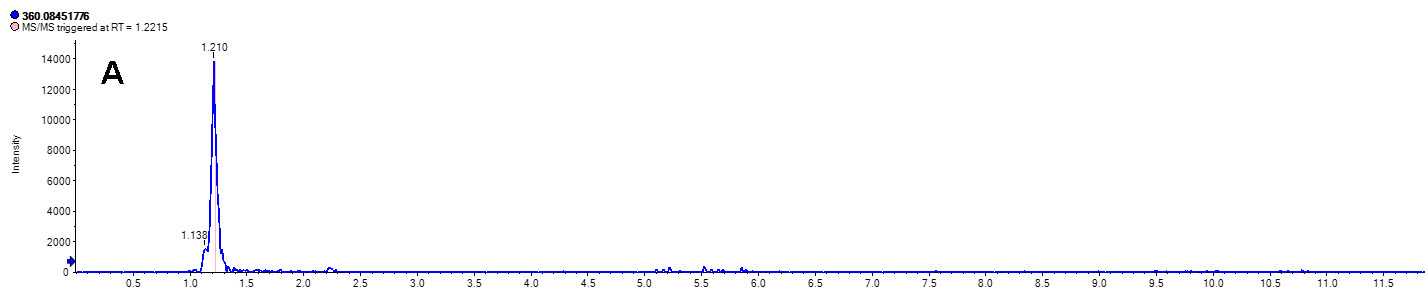


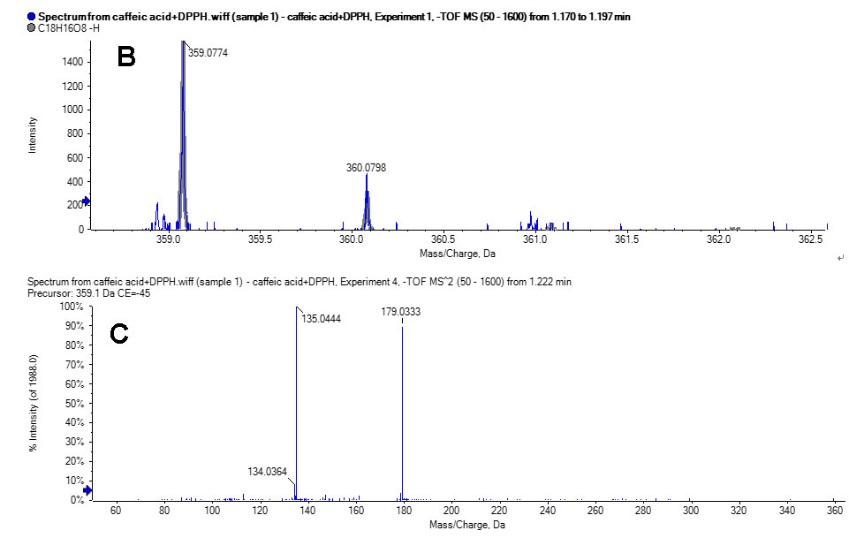


Fig. S2.15 Ion chromatogram of reaction product of **caffeic acid** dimer extracted by C_18_H_14_O_8._ The reaction was conducted by mixing caffeic acid and DPPH• (1:2, molar ratio) . The reaction mixture was incubated for 24h. The product mixture was analyzed using UPLC−ESI−Q−TOF−MS/MS technology. For the convenience of determination, the corresponding chemical formula of caffeic acid-caffeic acid dimer was extracted by the software. The corresponding chemical formula was C_18_H_14_O_8_ (caffeic acid is C_9_H_8_O_4._ Thus, the dimer should be C_18_H_14_O_8_).

As seen in the Fig. S2.15A, the spectra give a evident corresponding peak at 1.210 min.

The peak at 1.210 min yielded a molecular ion peak *m/z* 359-360 (Fig. S2.15B)

The molecular ion peak in Fig. S2.15B was further broken to give rise to secondary MS spectra *m/z* 179, 135 in Fig. S2.15C. In terms of literatures (Brand-Williams, W., M. E. Cuvelier, C. Berset, Use of a Free Radical Method to Evaluate Antioxidant Activity. Lebensm.-Wiss. u.-Technol, 1995. 28: p. 25-30; Foti, M.C., Antioxidant properties of phenols. J Pharm Pharmacol, 2007. 59(12): p. 1673-85), the pathway and MS spectra elucidation of one of RAF product can be described as the following:

**In a word, Fig. S2.15A, B, and C display that, caffeic acid happened dimerization reaction when mixed with DPPH•.**
