# Supplementary material for: Antioxidation and Cytoprotection of Acteoside and Its Derivatives: Comparison and Mechanistic Chemistry
Source: Molecules. 2018 Feb 23;23(2):498. doi: 10.3390/molecules23020498 (PMC6017589; doi:10.3390/molecules23020498)
Supplement: Supplementary file 1 [file molecules-23-00498-s001.zip › Suppls/Suppl. 3 Analysis Certificates.pdf]

西力生物
BBP BioBioPha
Natural Product Library for Drug Discovery

CERTIFICATE OF ANALYSIS

BBP No.: BBP00491

CAS No.: 61276-17-3

Chemical Name: Acteoside

Molecular Formula: $C_{29}H_{36}O_{15}$

Structure:

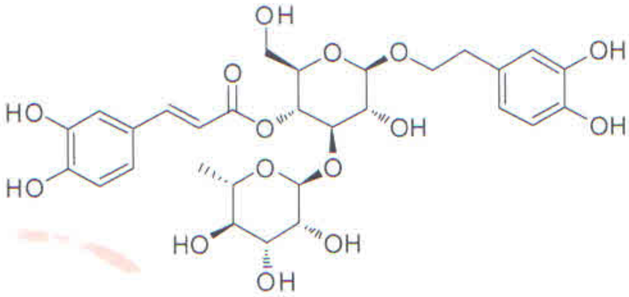

Purity: 95%

Appearance: Powder

Solvent: Methanol

Exact Weight: 5.1 mg

Storage: Store in a dark place under the temperature of 0−4 °C

Intended Use: For laboratory use only

Reference: H. Kobayashi, et al., Chem. Pharm. Bull., 1984, 32(8), 3009-3014

Warm Notice: When publishing, please cite as: ***chemical name*** was purchased from BioBioPha Co., Ltd. (Kunming, China)

Characterization Data Summary

Analytical Test	Results
Identification by ^{1}H-NMR	Consistent with the above structure
Purity tested by HPLC, ^{1}H-NMR	95%

Authorized Signature: Fei Wang

Date: 2016-11-2

BioBioPha Co.,Ltd.
2016.11.02
PASS

西力生物
BBP BioBioPha
Natural Product Library for Drug Discovery

PRODUCT QUALITY REPORT

Product Number: BBP00491

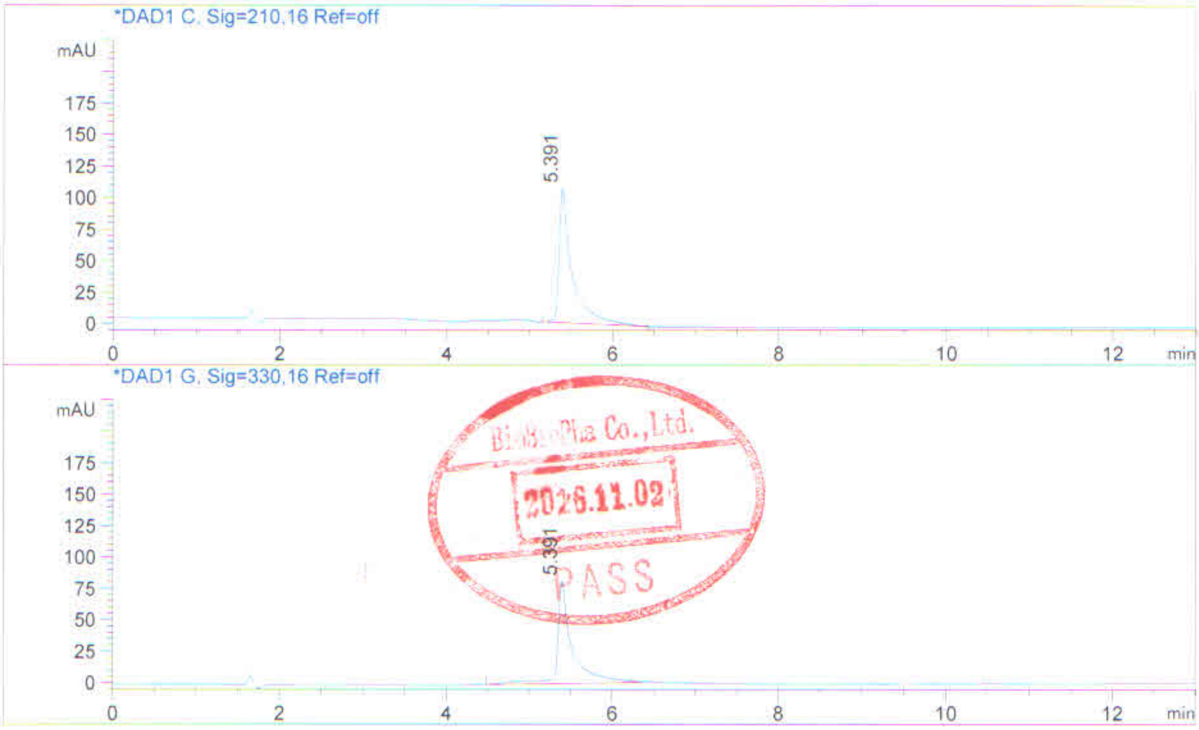

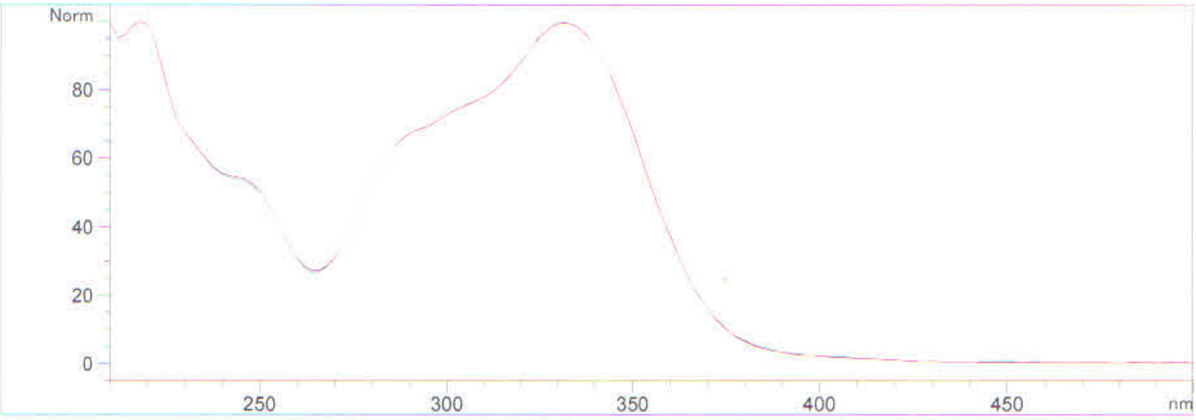

Agilent 1200 series HPLC system
Thermo Hypersil BDS C18 column (5 μm, 4.6 × 150 mm)
20%→100% MeOH in H_2O over 8 min followed by 100% MeOH to 13.0 min
1.0 ml/min, 25°C

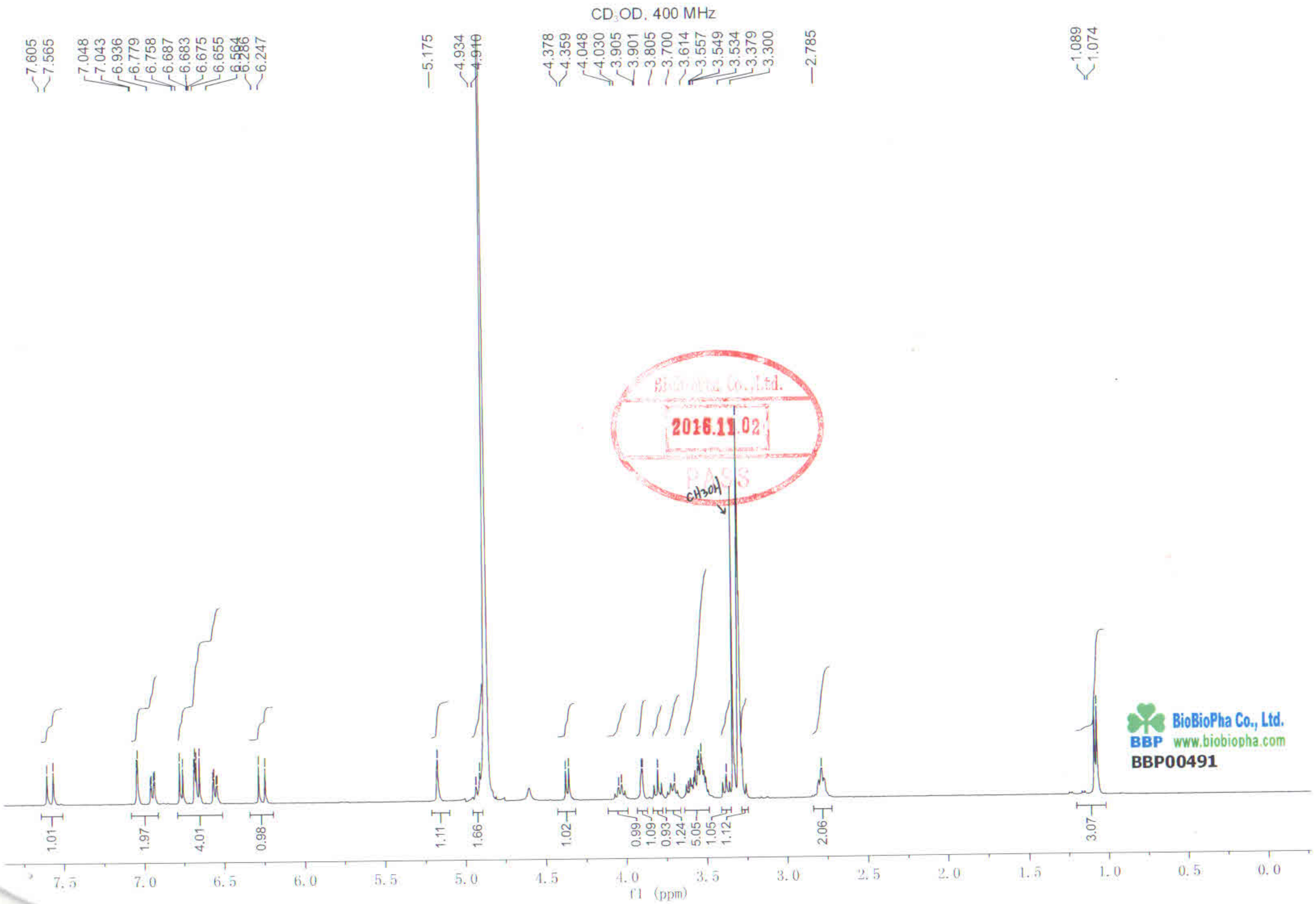

BioBioPha Co., Ltd.
BBP www.biobiopha.com
BBP00491

西力生物
BBP BioBioPha

Natural Product Library for Drug Discovery

CERTIFICATE OF ANALYSIS

BBP No.: BBP04578

CAS No.: 81525-13-5

Chemical Name: Forsythoside B

Molecular Formula: $C_{34}H_{44}O_{19}$

Structure:

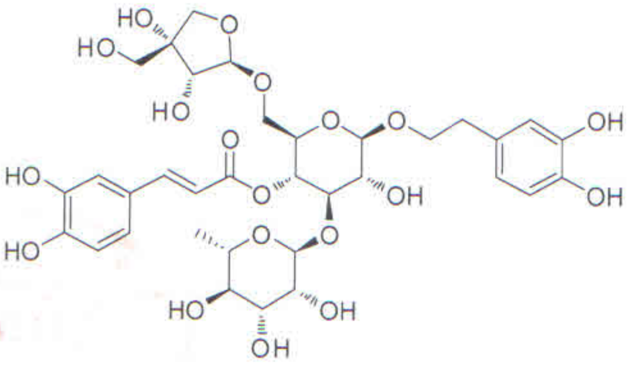

Purity: 97%

Appearance: Powder

Solvent: Methanol

Exact Weight: 5.1 mg

Storage: Store in a dark place under the temperature of 0−4 °C

Intended Use: For laboratory use only

Reference: I. Calis, Phytochemistry, 1992, 31(10), 3624-3626

Warm Notice: When publishing, please cite as: ***chemical name*** was purchased from BioBioPha Co., Ltd. (Kunming, China)

Characterization Data Summary

Analytical Test	Results
Identification by ^{1}H-NMR	Consistent with the above structure
Purity tested by HPLC, ^{1}H-NMR	97%

Authorized Signature: T.e. Wang

Date: 2016.11.2

BioBioPha Co., Ltd
2016.11.02
PASS

Tel.: +86 871 6521 7109 Fax: +86 871 6521 5563 E-mail: sales@mail.biobiopha.com

西力生物
BBP BioBioPha
Natural Product Library for Drug Discovery

PRODUCT QUALITY REPORT

Product Number: BBP04578

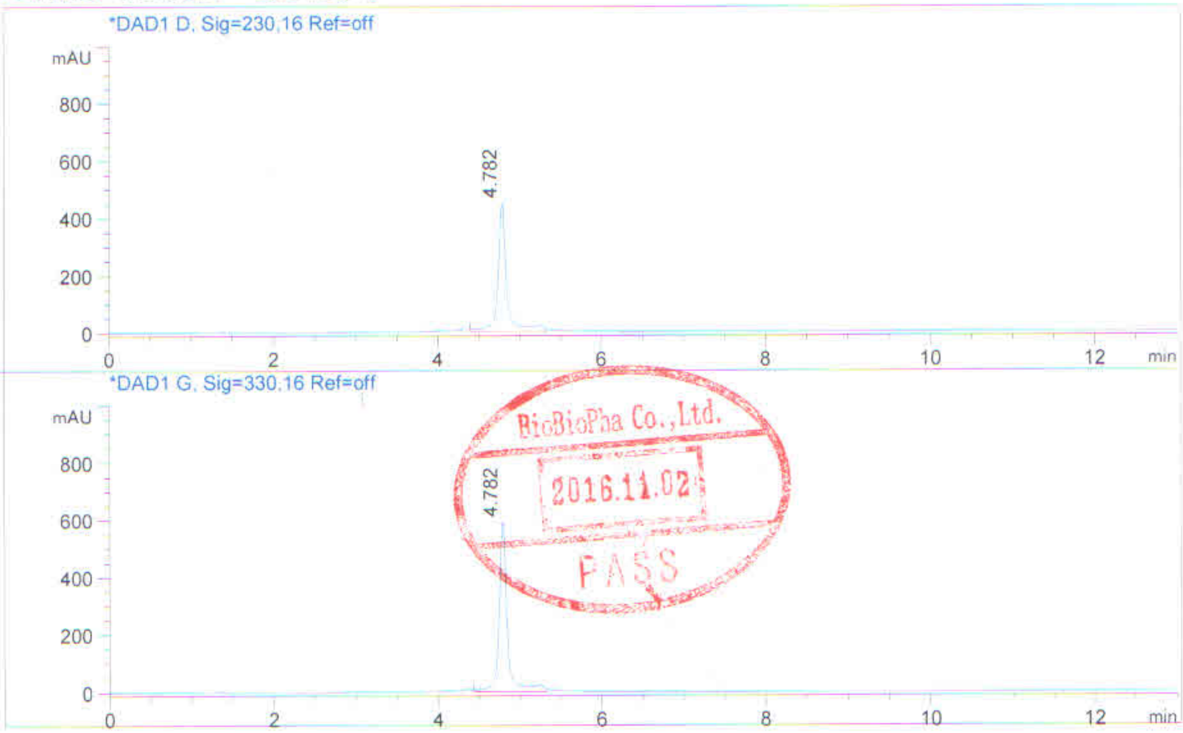

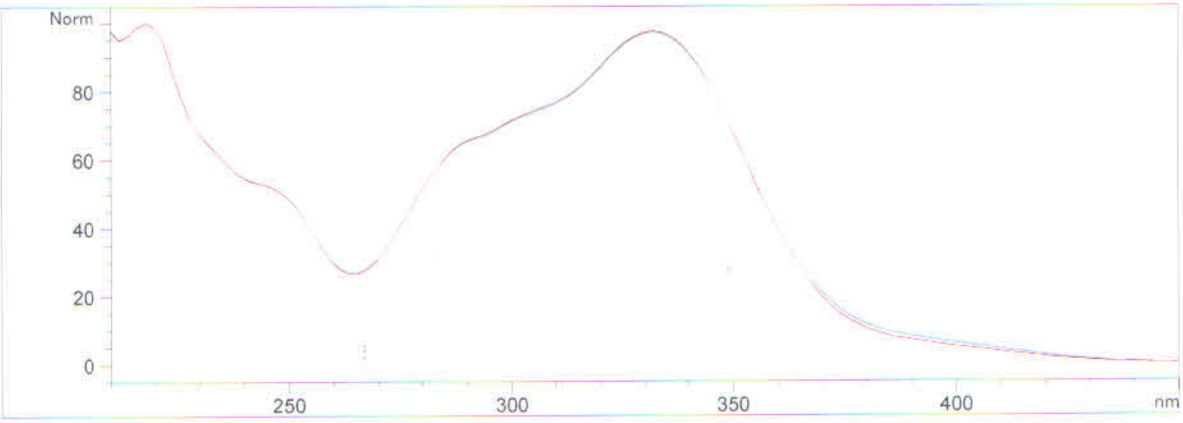

Agilent 1200 series HPLC system
Extend-C18 column (5 μm, 4.6 × 150 mm)
20%→100% MeOH in H_2O over 8.0 min followed by 100% MeOH to 13.0 min
1.0 ml/min, 20°C

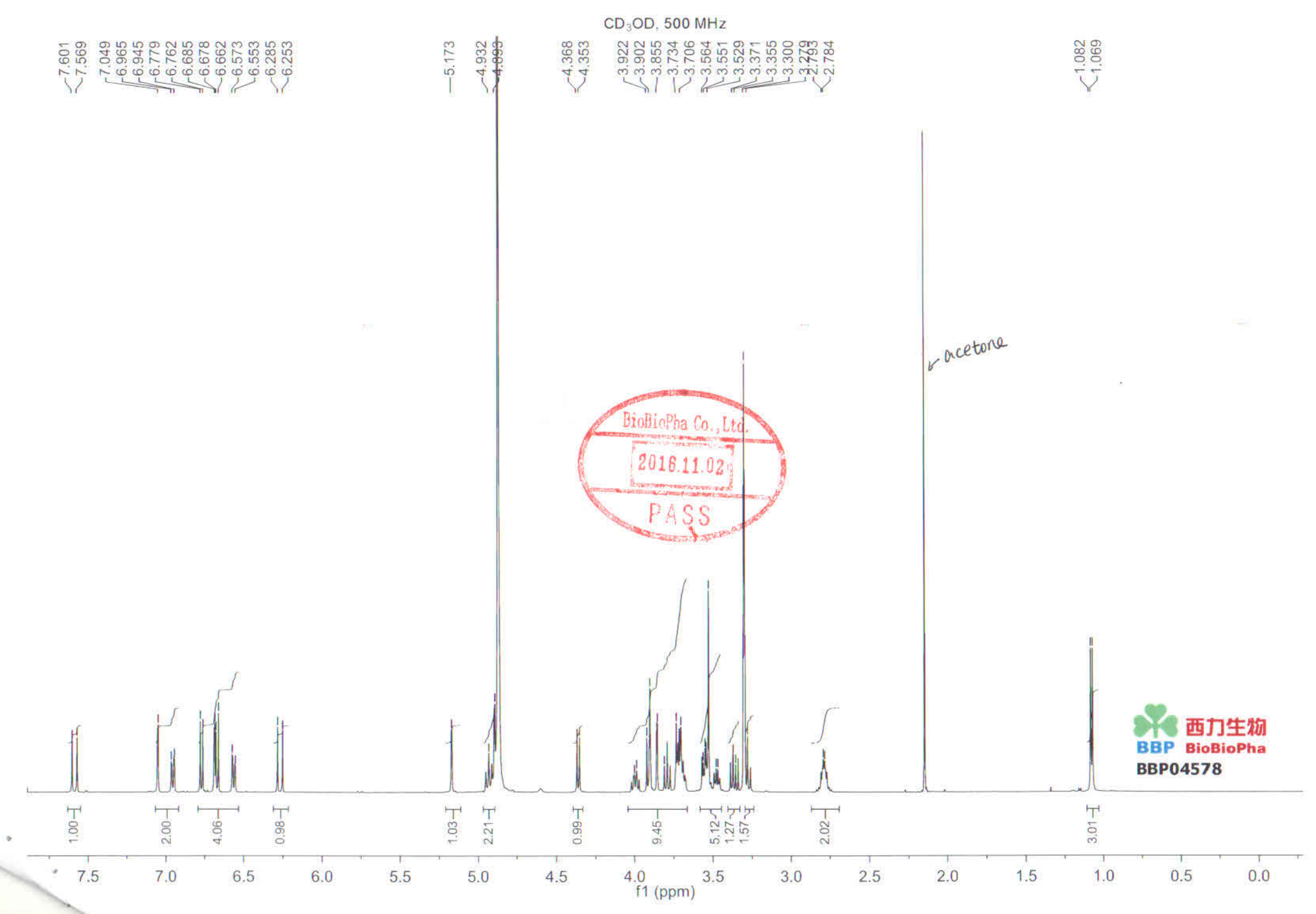

CD3OD, 500 MHz
7.601
7.569
7.049
6.965
6.945
6.779
6.762
6.685
6.678
6.662
6.573
6.553
6.285
6.253
5.173
4.932
4.893
4.368
4.353
3.922
3.902
3.855
3.734
3.706
3.564
3.551
3.529
3.371
3.355
3.300
3.279
2.793
2.784
1.082
1.069
acetone
BioBioPha Co., Ltd.
2016.11.02
PASS
1.00
2.00
4.06
0.98
1.03
2.21
0.99
9.45
5.12
1.27
1.57
2.02
3.01
7.5
7.0
6.5
6.0
5.5
5.0
4.5
4.0
3.5
3.0
2.5
2.0
1.5
1.0
0.5
0.0
f1 (ppm)
西力生物
BBP BioBioPha
BBP04578

poliumoside 0.5mg/mL

谱图文件:C:\浙大智达\N2000\样品\poliumoside.dat

报告时间：2017-12-13,22:34:57
积分方法:面积归一法

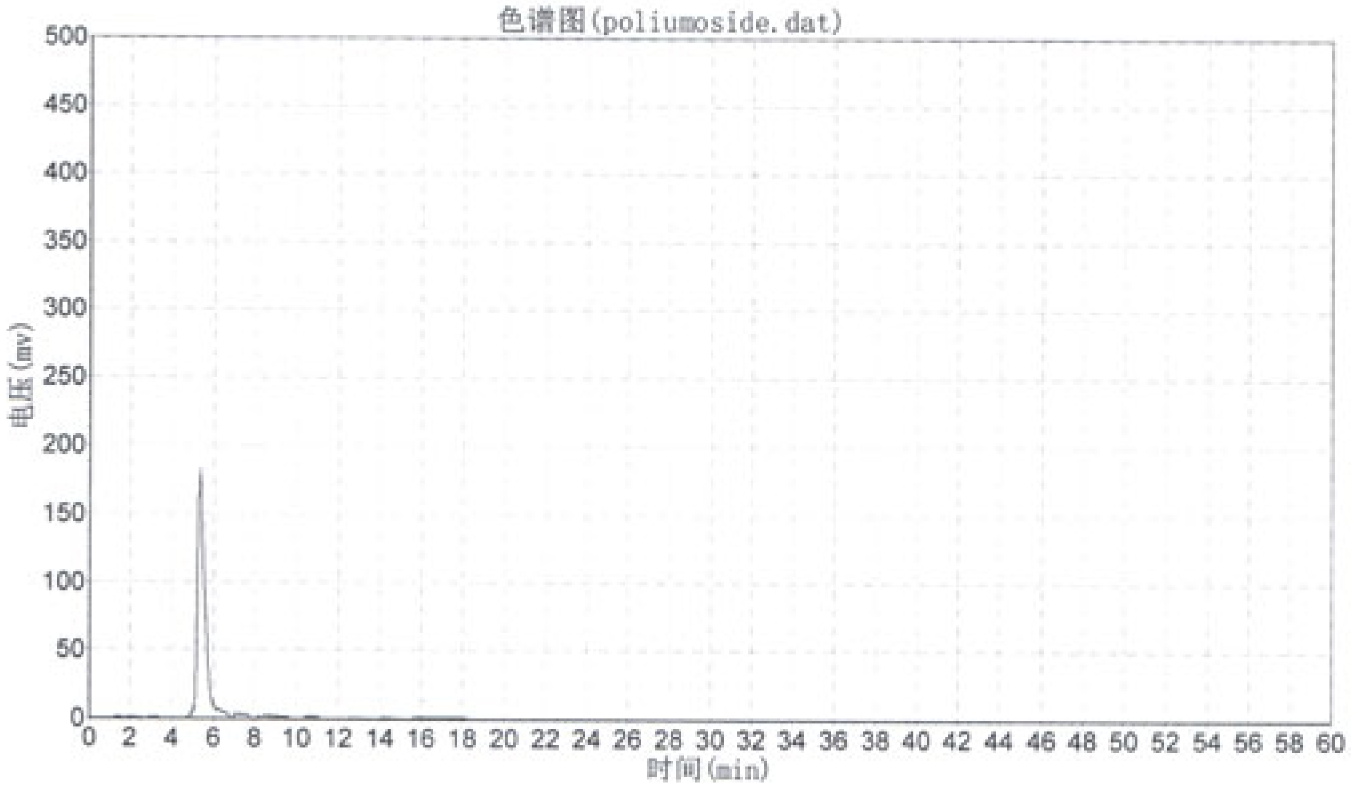

分析结果表

峰号	峰名	保留时间	峰高	峰面积	含量
1		4.973	8757.696	123533.969	2.7519
2		5.348	180670.422	4365471.500	97.2481
总计			189428.118	4489005.469	100.0000

HPLC system equipped with a UV30 detector (Syltech P510 HPLC, Los Angeles, USA)

Diamonsil C18 column (250 mm×4.6 mm, 5 μm, Dikma Ltd., China). Wavelenth 332 nm; Mobile MeOH：H2O=30:70

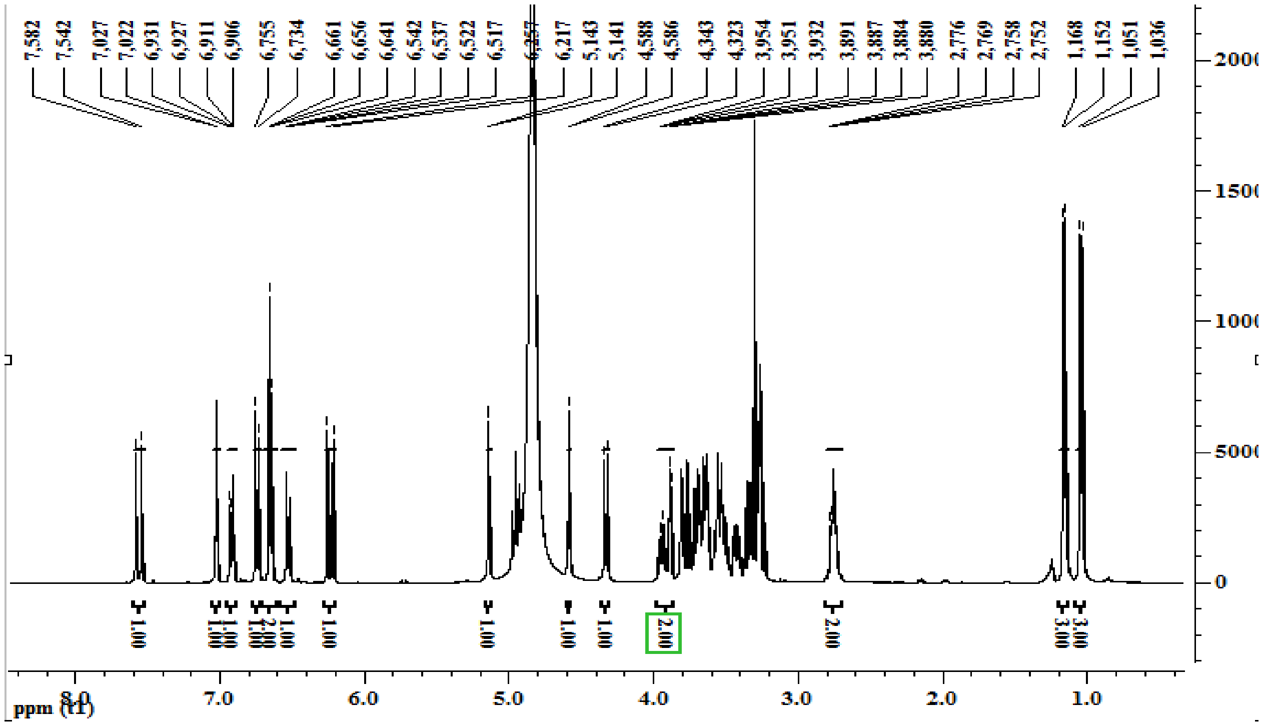

Fig.1 The ^{1}H NMR spectrum of poliumoside

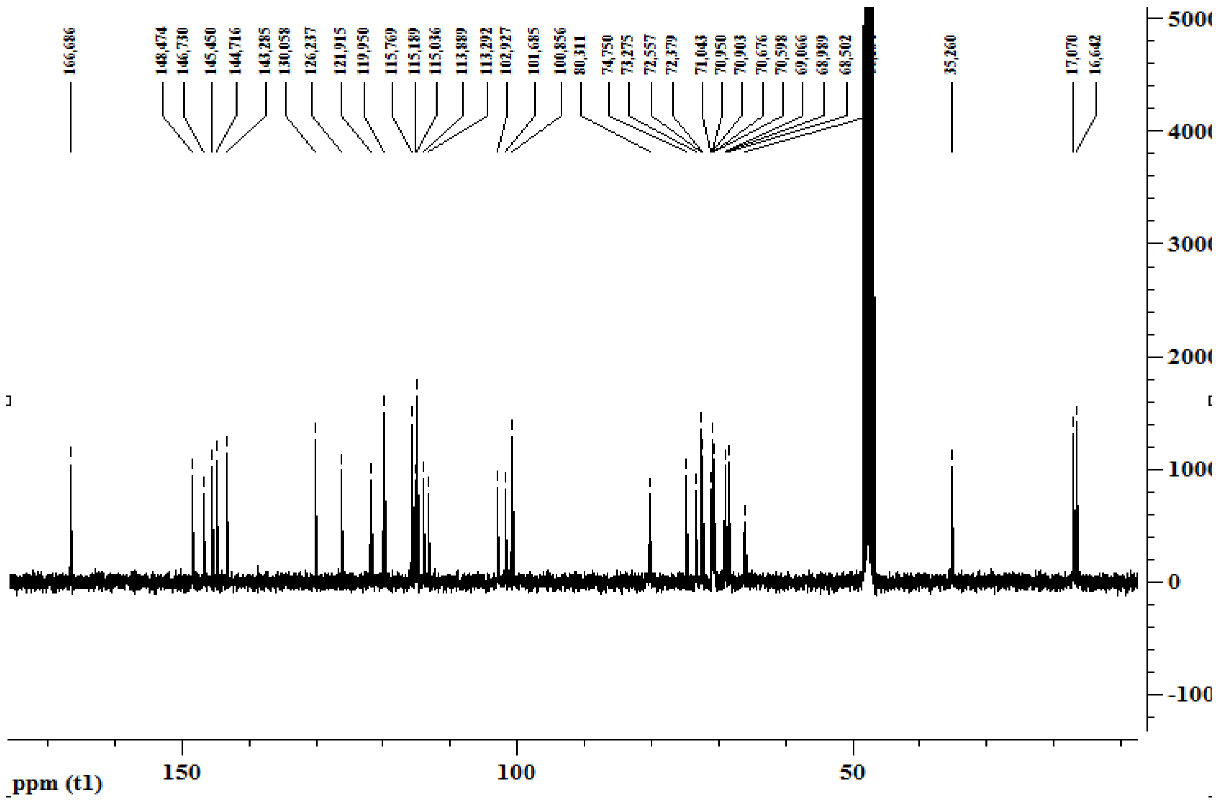

Fig.2 The 13C NMR spectrum of poliumoside

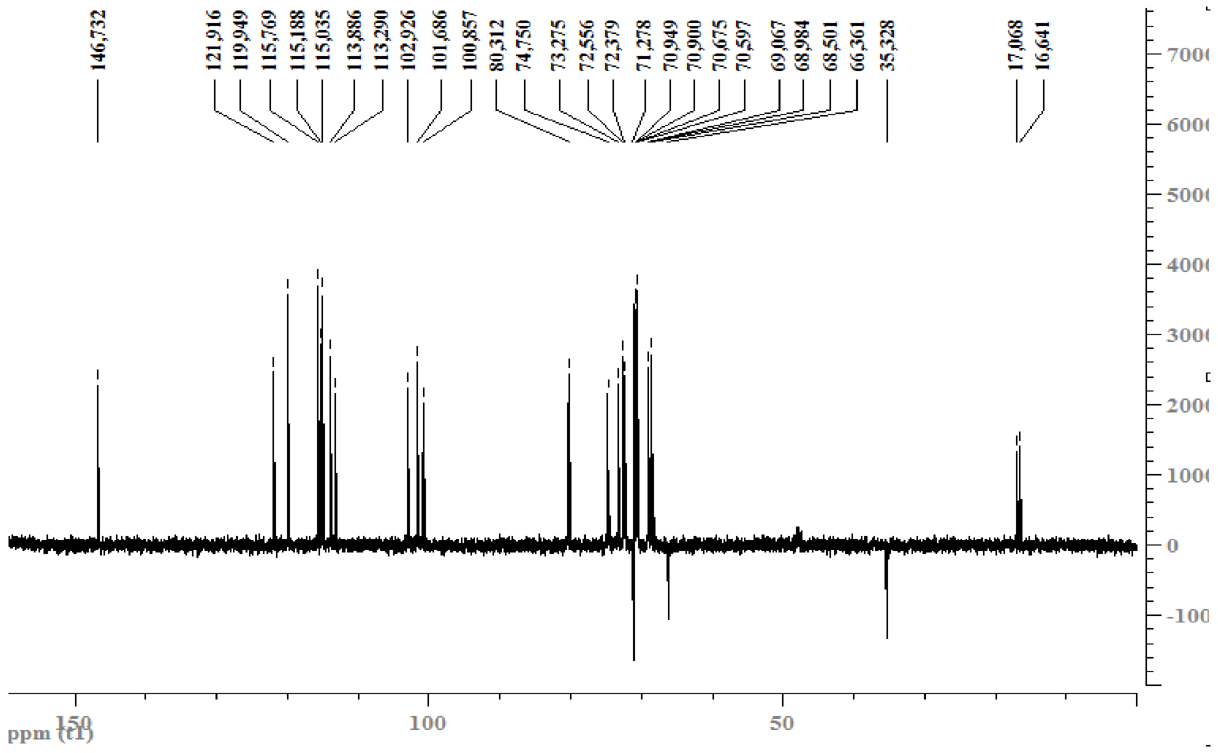

Fig.3 The ^{13}C NMR DEPT-135 spectrum of poliumoside
